# Transgenic expression of fungal accessory hemicellulases in *Arabidopsis thaliana* triggers transcriptional patterns related to biotic stress and defense response

**DOI:** 10.1371/journal.pone.0173094

**Published:** 2017-03-02

**Authors:** Alex Yi-Lin Tsai, Kin Chan, Chi-Yip Ho, Thomas Canam, Resmi Capron, Emma R. Master, Katharina Bräutigam

**Affiliations:** 1 Department of Cell and Systems Biology, University of Toronto, Toronto, Ontario, Canada; 2 Joint BioEnergy Institute, Lawrence Berkeley National Laboratory, Berkeley, California, United States of America; 3 Lunenfeld-Tanenbaum Research Institute, Mount Sinai Hospital, Toronto, Ontario, Canada; 4 Department of Biological Sciences, Eastern Illinois University, Charleston, Illinois, United States of America; 5 Department of Chemical Engineering & Applied Chemistry, University of Toronto, Toronto, Ontario, Canada; 6 Department of Biology, University of Toronto Mississauga, Mississauga, Ontario, Canada; Iowa State University, UNITED STATES

## Abstract

The plant cell wall is an abundant and renewable resource for lignocellulosic applications such as the production of biofuel. Due to structural and compositional complexities, the plant cell wall is, however, recalcitrant to hydrolysis and extraction of platform sugars. A cell wall engineering strategy to reduce this recalcitrance makes use of microbial cell wall modifying enzymes that are expressed directly in plants themselves. Previously, we constructed transgenic *Arabidopsis thaliana* constitutively expressing the fungal hemicellulases: *Phanerochaete carnosa* glucurnoyl esterase (*PcGCE*) and *Aspergillus nidulans* α-arabinofuranosidase (*AnAF54*). While the *PcGCE* lines demonstrated improved xylan extractability, they also displayed chlorotic leaves leading to the hypothesis that expression of such enzymes *in planta* resulted in plant stress. The objective of this study is to investigate the impact of transgenic expression of the aforementioned microbial hemicellulases *in planta* on the host arabidopsis. More specifically, we investigated transcriptome profiles by short read high throughput sequencing (RNAseq) from developmentally distinct parts of the plant stem. When compared to non-transformed wild-type plants, a subset of genes was identified that showed differential transcript abundance in all transgenic lines and tissues investigated. Intriguingly, this core set of genes was significantly enriched for those involved in plant defense and biotic stress responses. While stress and defense-related genes showed increased transcript abundance in the transgenic plants regardless of tissue or genotype, genes involved in photosynthesis (light harvesting) were decreased in their transcript abundance potentially reflecting wide-spread effects of heterologous microbial transgene expression and the maintenance of plant homeostasis. Additionally, an increase in transcript abundance for genes involved in salicylic acid signaling further substantiates our finding that transgenic expression of microbial cell wall modifying enzymes induces transcriptome responses similar to those observed in defense responses.

## Introduction

Plant cell walls are primarily composed of lignocellulose, which represents a structural complex of cellulose, hemicellulose, and lignin cross-linked through various covalent and non-covalent interactions [1]. They also form the bulk of plant biomass. Given its abundant and renewable nature, plant biomass is an attractive resource for biofuels, biomaterials and biochemicals. However, one barrier to the aforementioned lignocellulosic applications is the biological recalcitrance of plant cell walls, and in particular, resistance to enzymatic hydrolysis. This is due in part to the presence of lignin as a physical barrier that is thought to surround cellulose fibrils resulting in a multitude of non-specific molecular interactions, limited accessibility, and inhibition of enzymatic activities [2]. Such hindrance leads to an increase in enzyme loading, hydrolysis time, and the requirement of additional pretreatment procedures. In particular, the high cost of the enzymes required for lignocellulosic fractionation and hydrolysis remains a major hurdle that needs to be overcome for the adoption of economically viable lignocellulosic applications [3].

Microbes and other organisms encode an array of enzymes that can readily degrade polysaccharides and lignin in plant cell walls. Biological pretreatment takes advantage of these natural catalysts by treating the plant biomass with these organisms. White rot fungi, for example, degrade lignin and hemicellulose and can be used as pretreatment to improve subsequent enzymatic hydrolysis and saccharification of cellulose [4]. An alternative approach aims at the expression of microbial enzymes directly at the site of intended action, i.e. within plants themselves. For example, given their importance in cell wall degradation, various hemicellullases have been plausible candidates for transgenic expression *in planta* as a means to reduce biomass recalcitrance. To date, an array of xylanases, mannanases, and accessory hemicellulases has been expressed *in planta* yielding transgenic plants with improved feedstock traits such as increased carbohydrate content, decreased lignin content, reduced degree of lignocellulosic linkages, or improved saccharification [5–7]. A recent study also showed that the combined expression of two distinct microbial cell wall-degrading enzymes *in planta*, an endoxylanase and a feruloyl esterase, yielded in synergistic activities that further improved the cellulase digestibility of plant cell wall material [7].

In some cases, however, undesirable phenotypes that negatively impacted plant development have also been observed in the aforementioned transgenic plants, such as stunted growth, reduced plant biomass, delayed development and necrotic or chlorotic leaves [8–12]. While plant cell walls are critical for the structural integrity and support of the plant itself, they also provide a physical barrier against (a)biotic stresses; thus, plant cell walls have been implicated in plant immunity and biotic stress responses [13–15]. A successful invasion first requires potential microbial phytopathogens to penetrate the cell wall. Therefore, the plant cell wall has evolved to play critical roles in surveillance and other defense systems such as generation or perceptions of pathogen-associated or damage-associated molecular patterns (PAMP or DAMP) that refer to microbial parts (e.g. flagellin, chitin) or cell wall fragments as part of cell wall degradation. Even loss of acetyl or feruloyl substituents from the hemicellulose arabinoxylan is sufficient to initiate cell wall immunity [16,17]. External applications of microbial hemicellulases to leaves can act as an elicitor that triggers the expression of individual defense-related genes in plants [18–20]. Accordingly, it is important to understand the underlying molecular and regulatory roles that integrate cell wall structure and function into the framework of cellular and plant responses when employing cell wall engineering strategies. In particular, complex effects of transgenically expressed microbial cell wall modifying enzymes might escape detection when focusing on cell wall characteristics alone such as cell wall composition or extractability characteristics, yet they might have important implications for raw biomass production under controlled and field conditions.

Previously, it was demonstrated that the transgenic expression of *Phanerochaete carnosa* glucuronoyl esterase (PcGCE) in *Arabidopsis thaliana* resulted in altered cell wall architecture and improved xylan extractability [6]. At the same time, the transgenic plants displayed additional phenotypes such as yellowing of rosette leaves reminiscent of early senescing. A similar phenotype was also observed in arabidopsis expressing another fungal hemicellulase, an *Aspergillus nidulans* family 54 α-arabinofuranosidase (AnAF54) (S1 Fig). The aim of this study is to investigate the response of the plant host to constitutive expression of microbial hemicellulases *in planta* by examining the transcriptome of the transgenic *A*. *thaliana*.

## Materials and methods

### Plant cultivation

The transgenic *A*. *thaliana* lines expressing PcGCE (GenBank Accession: AFM93784.1) and AnAF54 (GenBank Accession: O74288) were constructed with *Agrobacterium*-mediated transformation and cultivated as previously described using the arabidopsis ecotype Columbia 0 (Col-0) as genetic background [6]. Two independent lines overexpressing PcGCE (*PcGCE-7* and *PcGCE-13*) and one line for AnAF54 were included in this study. Plant tissues from 52 plants per line were harvested at developmental stage 6.10 [21], which corresponded to the stage when 10% of flowers were produced or opened. To capture developmentally and chemically distinct lignocellulose material, primary inflorescence stems were then collected and divided into top and middle regions according to plant height [22]. The top portion corresponded to the top 25% of the stem and the mid portion corresponded to the region ranging from 25% to 75% of the total height measured from the top. For each transgenic line, stem samples from 50 plants were randomly divided into two pools to generate biological duplicates for RNA extraction and analysis.

### RNA isolation, cDNA synthesis, and Illumina sequencing

Plant RNA was extracted from stem samples using the RNeasy Plant Mini Kit (Qiagen, Hilden, Germany). Briefly, pooled plant stems were submerged in liquid nitrogen and ground to a fine powder using mortar and pestle. Approximately 100 mg of powder were used for RNA extraction, which was performed by following the manufacturer’s instructions with the inclusion of on-column DNase (Qiagen) digestion. The RNA concentrations were quantified using a NanoDrop 1000 spectrophotometer (Thermo Scientific, Waltham, USA). cDNA libraries were prepared using Illumina’s TruSeq RNA Sample Prep Kit v2 (Illumina, San Diego, USA). Briefly, 1 μg of high quality total RNA was used to generate the cDNA library according to Illunima's protocol, and the generated barcoded cDNA library had an average fragment size of 350–400 bp. Quality check of this barcoded library was performed with the Bioanalyzer (Agilent, Santa Clara, USA), and the cDNA in this library was quantified by qPCR on an Applied Biosystems 7900HT (Applied Biosystems, Foster City, USA) using KAPA SYBR FAST qPCR Kit Master Mix (2X) Universal (Kapa Biosystems, Woburn, USA). Qualitatively and quantitatively verified libraries were then loaded on a flowcell for cluster generation using Illumina’s c-Bot and TruSeq PE Cluster Kit v3 (Illumina). Sequencing was carried out on an HiSeq2000 with the TruSeq SBS Kit v3 (pair-ended 200 cycles, Illumina). 100 bp pair-end reads were generated. The real-time base call (.bcl) files were converted to fastq files using CASAVA 1.8.2 (Illumina) for further sequence analyses.

### Sequence processing and differential expression

Reads were aligned to the latest arabidopsis genome assembly (TAIR10, Ensembl, release September 2010) with TopHat version 2.0.8 and Bowtie version 2.1.0 with minimum and maximum intron size set to 20 and 4000 bases, respectively [23,24]. Sorting and indexing of the resulting BAM (binary alignment/map) files was done using SAMtools version 0.1.18 [25]. Read counts for each gene were obtained with htseq-count version 0.6.1 using the sorted SAM (sequence alignment/map) files as input with the settings stranded = no, a = 10, and mode = union [26].

Differential transcript abundance was determined based on count data using the edgeR package version 3.12.0 in R [27]. After applying an intensity filter of at least two cpm (counts per million) in at least two samples 17,682 genes were included in the analysis. The following package functions were used in conjunction with a manually defined sum-to-zero contrast matrix: estimateDisp, glmFit and glmLRT with robust set to TRUE. Using edgeR functionalities, GLM (generalized linear model) likelihood ratio tests [27] were performed, and genes with an FDR < 0.05 were considered to exhibit statistically significant differences in transcript abundance for the contrast (comparison) under consideration.

### Bioinformatic tools

Gene ontology (GO) slim terms were annotated using the TAIR bulk GO annotation retrieval tool, and enrichment was calculated using the phyper function in R using the distribution of GO terms among all confidently detectable genes (17,682 after application of an intensity filter) as background [28]. The Plant Gene Set Enrichment Analysis (PlantGSEA) was used to identify gene enrichment in GO ontology [29], KEGG pathways and gene expression data from the literature. All raw sequencing data of samples were uploaded to the NCBI Sequence Read Archive (http://www.ncbi.nlm.nih.gov/sra); accession number SRP081029.

### Quantitative RT-PCR

Seven salicylic acid-related genes with differential gene expression between transgenic and wild-type plants were selected for quantitative RT-PCR (qRT-PCR) validation. Both, top stem and mid stem samples were analyzed. Total RNA was extracted from frozen, ground stem material using the RNeasy Plant Mini Kit (Qiagen, Hilden, Germany), and 1 μg of total RNA was reverse transcribed using the Bio-Rad iScript cDNA Synthesis kit (Bio-Rad, Hercules, USA) according to the manufacturer’s instructions. The 10-fold diluted cDNA was used as a template. The qRT-PCR reaction was run on an ABI 7500 Fast Real-Time PCR System (Applied Biosystems) using Bio-Rad SsoAdvanced Universal SYBBR Green Supermix (Bio-Rad) with the following settings: 95°C for 30 sec, 40 cycles of 95°C for 10 sec and 60°C for 30 sec. Relative transcript abundance was calculated according to Pfaffl (2001) [30] using *ACTIN* as reference. Primer sequences are listed in S1 Table.

## Results and discussion

### Different transgenic arabidopsis lines expressing microbial cell wall modifying enzymes show marked similarities in their transcriptome responses

**Fig 1:**
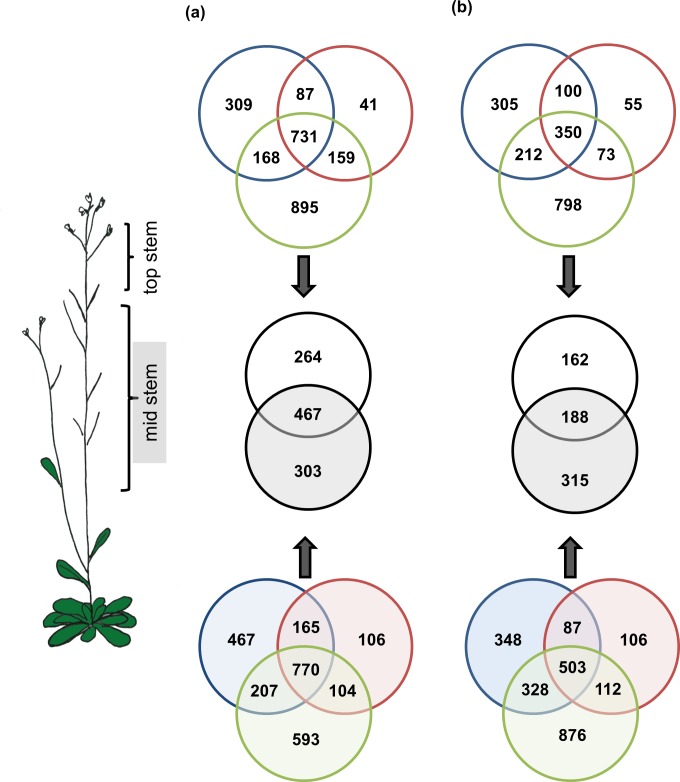
**Venn diagrams for genes with (a) increased and (b) decreased transcript abundance in transgenic plants relative to non-transformed wild-type plants**. The blue, red, and green circles represent the *PcGCE-7*, *PcGCE-13*, and *AnAF54* transgenic lines, respectively; while the open and shaded circles represent top and mid stems, respectively. A black circle represents the intersect of all three circles in each Venn diagram; while the overlap between the open and shaded black circles represent the core set genes.

For transcritpome analyses, two independently transformed arabidopsis lines constitutively expressing *Phanerochaete carnosa* glucuronoyl esterase and one line expressing *Aspergillus nidulans* α-arabinofuranosidase were selected. The two glucuronoyl esterase lines, *PcGCE-7* and *PcGCE-13*, show an altered cell wall architecture as the result of the transgene expression [6]. Meanwhile, *AnAF54*, the line expressing α-arabinofuranosidase, was selected as it displayed phenotypic similarities to the *PcGCE* lines with regards to early leaf yellowing (S1 Fig). Transcriptomes from non-transformed wild-type arabidopsis Col-0 were also included for comparison. In all cases, two developmentally distinct stem regions were studied: young elongating stem (top stem) and a stem region representing both intermediate and mature stem regions (mid stem), which were selected based on the height of the plants [22,31,32]. Top stem samples represent the apical 25% of the main plant stem (flowers removed) while mid stem samples represent the central 50% the stem (Fig 1). The two stem regions were selected to differentiate the formation of primary and secondary cell walls. Since the fungal hemicellulases selected in this study for transgenic expression are cell wall modifying enzymes, their catalytic action and any potential subsequently induced response may be correlated with the process of cell wall formation. Stems represent the tissue of interest in many plant cell wall engineering strategies. Hence, the top and mid stem tissues were selected for the transcriptome analysis, as they are enriched in cell types that are abundant in primary and secondary cell wall, respectively.

For each transgenic line and tissue, differential transcript abundance relative to the matching tissue in wild-type plants was calculated (S2 Table) and a false discovery rate (FDR) of < 0.05 was considered for statistical significance. A list of genes with significant increase in transcript abundance relative to wild-type and a list of genes with significant decrease in transcript abundance relative to the wild-type were generated for each transgenic line and tissue. These gene lists were then compared with each other. This comparison revealed that 731 and 770 genes showed increased transcript abundance in all transgenic lines relative to the wild-type control in top and mid stems, respectively (Fig 1A). Similarly, 350 and 503 genes were characterized by decreased transcript abundance in all transgenic lines in top and mid stem samples, respectively (Fig 1B).

**Fig 2:**
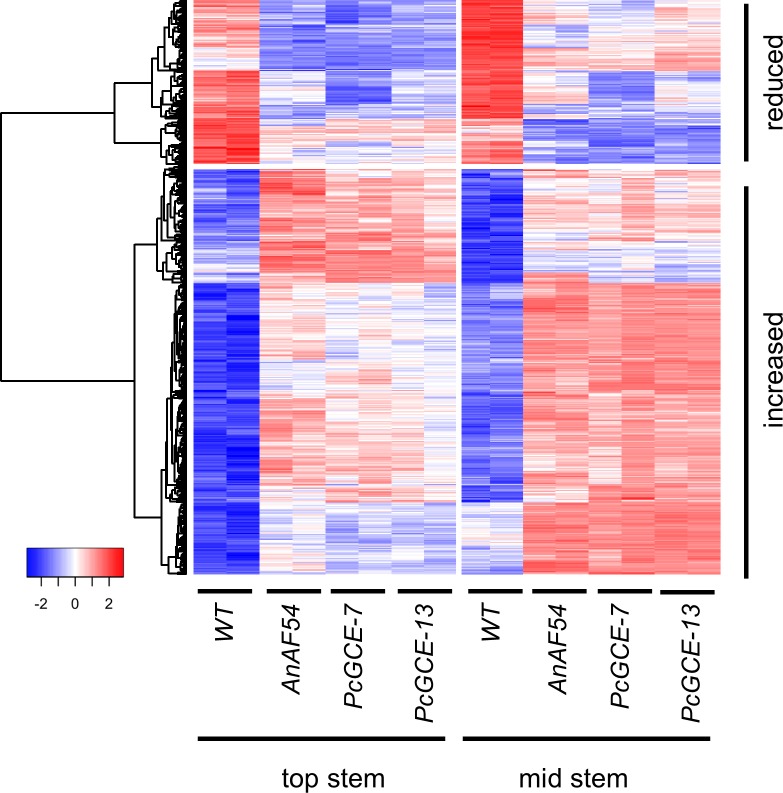
Core set of genes with differential expression in stem tissue of transgenic arabidopsis lines overexpressing fungal carbohydrate-active enzymes. The heat map represents relative transcript abundance of genes that differ significantly in each of the three transgenic lines (*AnAF54*, *PcGCE-7*, and *PcGCE-13*) relative to the wild-type in both, top stem and mid stem tissue (n = 655, FDR < 0.05). Genes with significantly lower transcript abundance in transgenic lines when compared with non-transformed wild-type are marked with “reduced” (n = 188) and gene with significantly higher transcript abundance are marked with “increased” (n = 467). Data are row normalized.

Closer inspection further revealed a high degree of overlap between differentially expressed genes in top stem and in mid stem: 467 genes had increased transcript abundance in all transgenic lines regardless of developmental stage and genotype (Fig 1A). A similar trend was also observed for genes with decreased transcript abundance, and a set of 188 genes with reduced expression in all transgenic lines and tissues was identified (Fig 1B). Herein, we refer to these 467 and 188 uniformly up and down regulated genes as the “core set” with altered expression profiles in all genotypes and tissues (Fig 2, S2 Fig, S3 Table). The comparison of gene lists also enabled identification of genes that were differentially regulated in response to a specific microbial transgene and/or its function, which will be discussed later in the manuscript.

### Transgenic arabidopsis exhibit transcriptional patterns related to stress and defense responses

**Fig 3:**
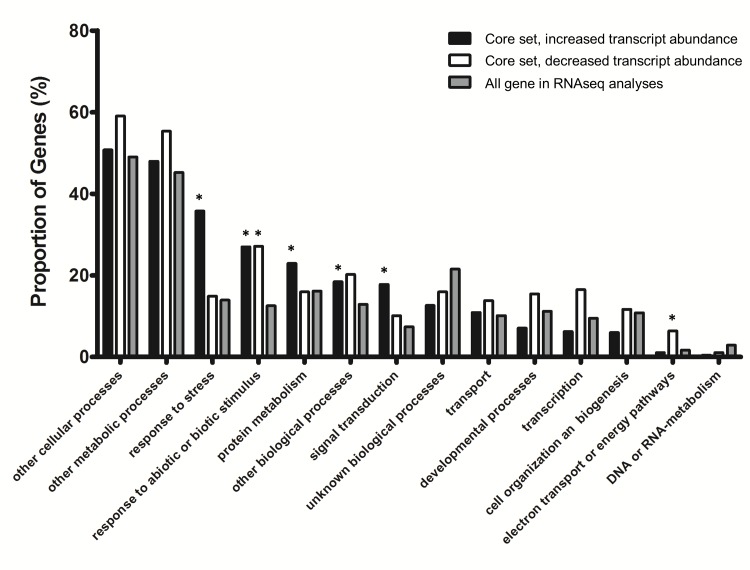
Gene ontology (GO) slim term enrichment of the core set genes for biological processes. GO slim terms were annotated using the TAIR bulk GO annotation retrieval tool. The proportion of genes in each GO slim category in the core set of genes was compared to that expected in the background (all arabidopsis genes that were detected as expressed in the experiment (S1 Table)). Asterisks indicate statistical significance (P < 0.05). Black and open bars represent genes with increased and decreased transcript abundance, respectively; while grey bars indicate the gene proportion in each GO slim term for all expressed arabidopsis genes (background).

**Fig 4:**
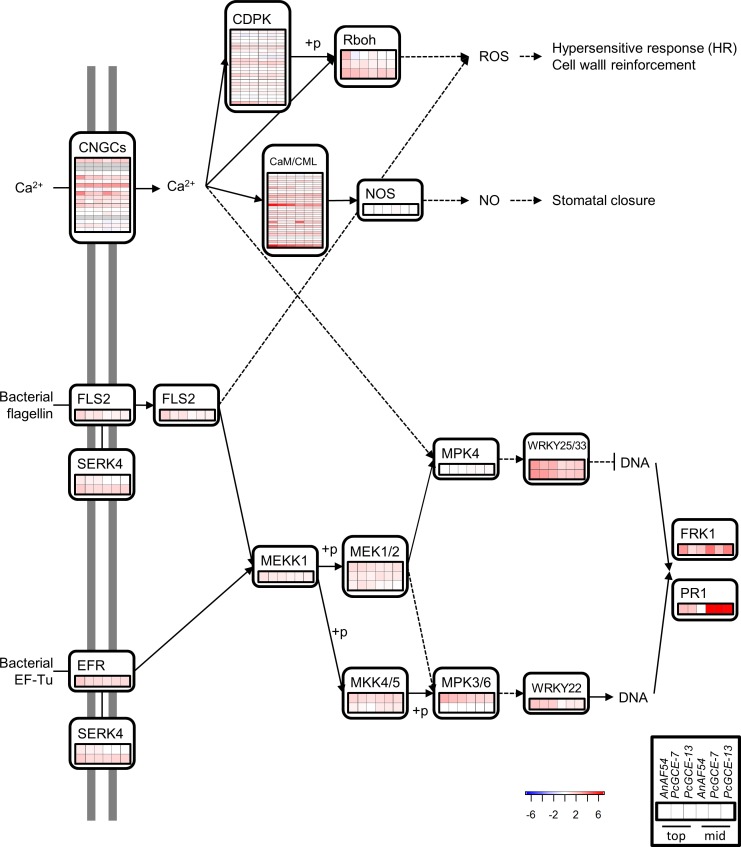
Plant-pathogen interaction pathway (KEGG ath04626). The pathway schematic was adapted from the KEGG database with permission [64,65]. The heat maps illustrate differential transcript accumulation in transgenic lines relative to wild-type for both top and mid stem samples. The color corresponds to log_2_ fold change in transcript accumulation. Rows in each heat map represent genes and the order of genes corresponds to the order given in S4A Table.

**Fig 5:**
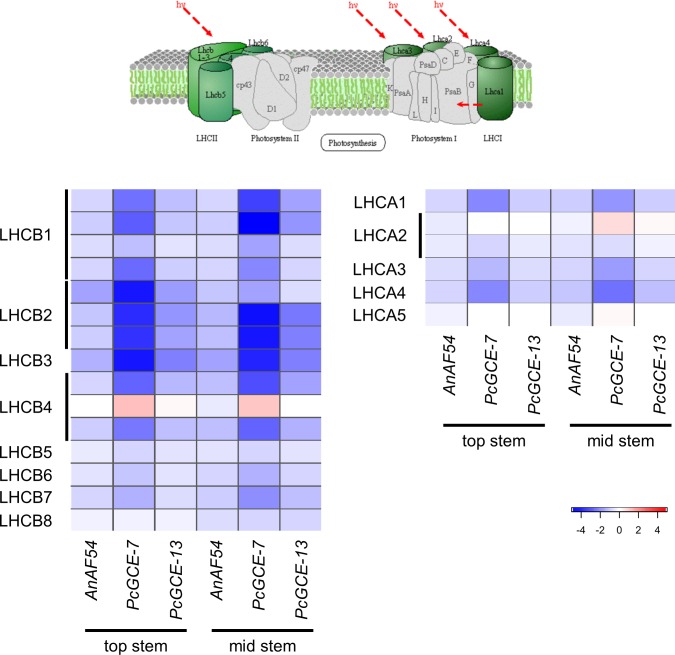
Photosynthesis—antenna protein pathway (KEGG ath00196). The pathway schematic was modified from the KEGG database with permission [64,65]. The heat maps illustrate differential transcript accumulation in transgenic lines relative to wild-type for both top and mid stem samples. The color corresponds to log_2_ fold change in transcript accumulation. Rows in each heat map represent genes and the order of genes corresponds to the order given in S4B Table.

To interpret the biological context behind the expression profiles, a series of gene enrichment analyses was performed, and the molecular function and biological role of the core set genes was investigated in greater detail (Figs 3–5; S3 Fig). Gene Ontology (GO) slim enrichment analyses of the core set revealed a strong enrichment for functions in response to stress (166; GO: 006950) and response to abiotic and biotic stress (125; GO: 009628 and 009607) (Fig 3). Moreover, the Plant Gene Set Enrichment Analysis (PlantGSEA) identified 20 genes in the core set with increased transcript abundance to be involved in the plant-pathogen interaction pathway (KEGG: ATH04626; S4A Table). 19 of the 20 genes (with exception of AT1G12220; RPS5) were able to map onto part of the plant-pathogen interaction pathway map (Fig 4). Both analyses agreed that the transgenic plants exhibit characteristics of stress or defense responses as the result of heterologously expressing microbial cell wall modifying enzymes. Upon closer inspection of the above mentioned pathway (KEGG: ATH04626), a number of genes such as membrane receptors (e.g. CNGC, SERK4), components of mitogen activated protein kinase (MAPK) cascades (e.g. MKK2), transcription factors (e.g. WRKY25), and regulatory proteins (e.g. CML) all showed increased transcript abundance in the transgenic plants regardless of tissue or transgene expressed. In addition, the transcript abundance for a subset of genes in this pathway such as MAPK cascade component (e.g. MKK, MPK) and downstream response genes (e.g. PR1, FRK1) was increased in all transgenic lines in one of the two tissues examined. Taken together, there appeared to be a clear and uniform tendency for increased transcript abundance in transgenic lines for genes involved in defense and biotic stress responses.

On the other hand, the PlantGSEA identified seven genes from the core set with reduced transcript abundance to be involved in the photosynthesis—antenna proteins pathway (KEGG: ATH00196; Fig 5; S4B Table), more specifically, genes coding for components of the light-harvesting complexes (LHC). LHC play crucial roles in photosynthesis. They bind chlorophyll and/or carotenoids that absorb photons and transfer light energy to the reaction centers of the photosystems. A reduction in LHC transcript abundance might point to a redirection of plant resources towards stress and defense responses at the expense of light harvesting complexes, and might also relate to the leaf yellowing phenotype that was observed in the transgenic plant rosettes. It has previously been shown that LHC expression is tightly regulated in response to environmental cues [33]. It has also been demonstrated that chlorophyll can be degraded in response to stress [34]. The interrelation between chlorophyll and LHC protein content might also provide a link between transgene expression and the early leaf yellowing observed in the transgenic lines included in this study. Another member of the “core set”, the pathogen-induced transcription factor WRKY40 showed, intriguingly, an increase in transcript abundance in the transgenic lines investigated in this study. This transcription factor has been characterized to repress LHC expression [33], and the observed patterns for WRKY40 provide a potential link/explanation for the reduction of LHCP in the transgenic plants.

### Defense response patterns associated with salicylic acid are detected in transgenic plants

Plant hormones such as abscisic acid, jasmonic acid, or salicylic acid are key players in various plant stress responses and defense mechanisms. Therefore, transcript levels of genes coding for enzymes associated with hormone metabolism were analyzed in greater detail. While some key enzymes involved in hormone biosynthesis such as ABA1, LOX, or ACS showed ubiquitous increase in transcript abundance in all genotypes and tissues examined, insufficient evidence was present to suggest transcriptional activation of entire hormonal biosynthesis pathways that may lead to increased hormone level in the transgenic plants (S4 Table). However, a number of genes involved in salicylic acid signaling pathways show increased transcript abundance in the transgenic plants. Genes involved in its upstream signaling (e.g. EDS1, PAD4, NDR1), positive feedback loop (e.g. SAG101), downstream signaling, (e.g. NPR1, NIMIN1, NIMIN3); induced defense genes (e.g. PR1, PR2, PR5), and transcription factors that regulate downstream responses to salicylic acid (e.g. WRKY transcription factors) [35] all showed increased transcript abundance in at least one tissue across all examined genotypes (Table 1). Such distinct expression patterns were visibly absent from downstream responses to other hormones (S5 Table**)**. In addition, PlantGSEA revealed enrichment of six salicylic acid-related GO terms among the core set genes (GO: 009697, 009696, 009751, 009863, 071446 and 009862) and to a lesser degree, three ethylene related GO terms (GO: 009723, 009692, and 009693). These results point towards an association of salicylic acid with the observed transcriptome profiles in the transgenic plants studied, including the specific induction of genes related to biotic stress and defense responses. The transcript abundance of seven salicylic acid-related genes was also determined by qRT-PCR. It confirmed the clear induction of these genes in the transgenic plants and also validated the quality of the RNAseq analyses (S4 Fig).

**Table 1:** Relative fold change of genes involved in salicylic acid signaling.

Gene Name	Top						Mid						Protein
	*AnAF54*		*PcGCE-7*		*PcGCE-13*		*AnAF54*		*PcGCE-7*		*PcGCE-13*		
	LogFC	FDR	LogFC	FDR	LogFC	FDR	LogFC	FDR	LogFC	FDR	LogFC	FDR	
AT3G48090	**1.663**	**0.001**	**1.718**	**0.001**	**1.242**	**0.037**	**1.834**	**0.000**	**1.899**	**0.000**	**1.594**	**0.003**	EDS1
AT3G52430	**2.376**	**0.000**	**2.027**	**0.000**	**1.742**	**0.000**	**1.256**	**0.001**	**1.016**	**0.011**	0.758	0.099	PAD4
AT3G20600	**2.933**	**0.000**	**2.460**	**0.000**	**2.260**	**0.000**	**1.952**	**0.002**	**2.379**	**0.000**	**2.421**	**0.000**	NDR1/MAPKKK
AT1G64280	**0.821**	**0.000**	**0.865**	**0.000**	**0.774**	**0.000**	**0.695**	**0.001**	**0.606**	**0.006**	**0.780**	**0.000**	NPR1
AT1G02450	**3.034**	**0.002**	**3.203**	**0.002**	**2.748**	**0.012**	**3.975**	**0.000**	**3.630**	**0.001**	**3.443**	**0.002**	NIMIN1
AT5G14930	**1.644**	**0.000**	**1.187**	**0.000**	**1.037**	**0.000**	**1.279**	**0.000**	**1.411**	**0.000**	**1.159**	**0.000**	SAG101
AT3G25070	-0.036	0.966	0.330	0.671	0.140	0.887	0.085	0.915	0.264	0.730	0.406	0.621	RIN4
AT2G14610	2.499	0.429	2.058	0.556	0.000	1.000	**12.004**	**0.000**	**7.009**	**0.000**	**6.757**	**0.000**	PR1
AT3G57260	**6.923**	**0.000**	**3.684**	**0.000**	**3.077**	**0.000**	**7.182**	**0.000**	**4.547**	**0.000**	**4.627**	**0.000**	PR2
AT1G75040	**2.784**	**0.000**	**1.803**	**0.000**	**1.539**	**0.000**	**5.547**	**0.000**	**3.582**	**0.000**	**4.060**	**0.000**	PR5
AT3G25882	**3.625**	**0.000**	**3.235**	**0.000**	**2.850**	**0.000**	**3.989**	**0.000**	**3.417**	**0.000**	**3.716**	**0.000**	NIMIN2
AT1G09415	-0.051	0.919	0.378	0.340	0.299	0.520	0.189	0.656	0.538	0.130	**0.710**	**0.038**	NIMIN3
AT5G65210	**-0.764**	**0.018**	-0.274	0.531	-0.245	0.618	**-0.781**	**0.008**	-0.465	0.175	-0.445	0.243	TGA1
AT5G10030	-0.790	0.226	-1.021	0.127	-0.486	0.581	-0.824	0.190	-0.400	0.613	-0.368	0.700	TGA4
AT5G06950	-0.201	0.545	-0.022	0.964	-0.160	0.711	0.097	0.786	-0.170	0.638	-0.116	0.807	TGA2
AT3G12250	-0.191	0.684	-0.315	0.504	-0.276	0.609	-0.187	0.673	-0.464	0.239	-0.322	0.510	TGA6
AT1G22070	**0.883**	**0.000**	0.201	0.586	0.533	0.074	**0.818**	**0.001**	0.244	0.453	**0.552**	**0.045**	TGA3
AT1G02930	**4.030**	**0.000**	1.551	0.094	**2.375**	**0.006**	**3.672**	**0.000**	**2.350**	**0.003**	**3.350**	**0.000**	GST6
AT4G31800	**3.123**	**0.000**	**2.580**	**0.000**	**2.421**	**0.001**	1.341	0.065	**1.683**	**0.019**	**1.527**	**0.048**	WRKY18
AT4G23810	**3.619**	**0.000**	**2.865**	**0.003**	**2.585**	**0.011**	**3.190**	**0.000**	**3.335**	**0.000**	**3.297**	**0.000**	WRKY53
AT2G40750	**4.877**	**0.000**	**4.884**	**0.000**	**4.465**	**0.000**	**3.433**	**0.000**	**3.992**	**0.000**	**3.824**	**0.000**	WRKY54
AT3G56400	**3.347**	**0.001**	**3.381**	**0.001**	**3.098**	**0.005**	**2.867**	**0.006**	**3.124**	**0.003**	**3.175**	**0.003**	WRKY70
AT3G01080	**3.291**	**0.000**	**3.333**	**0.000**	**3.263**	**0.000**	**2.456**	**0.000**	**2.725**	**0.000**	**2.864**	**0.000**	WRKY58
AT2G25000	**1.255**	**0.006**	0.896	0.088	0.682	0.276	0.800	0.099	0.842	0.092	0.595	0.330	WRKY60
AT4G24240	**2.120**	**0.000**	**2.220**	**0.000**	**2.149**	**0.000**	**1.005**	**0.003**	**1.355**	**0.000**	**1.463**	**0.000**	WRKY7
AT4G31550	1.813	0.175	1.444	0.349	1.382	0.414	1.342	0.336	1.052	0.504	1.108	0.535	WRKY11
AT2G24570	**1.679**	**0.000**	**1.420**	**0.002**	**1.092**	**0.042**	**1.221**	**0.008**	0.761	0.152	0.624	0.316	WRKY17
AT5G22570	**5.468**	**0.000**	**3.968**	**0.006**	**3.527**	**0.037**	**7.917**	**0.000**	**5.522**	**0.016**	**6.291**	**0.002**	WRKY38
AT1G14410	0.061	0.888	0.199	0.633	-0.050	0.931	0.614	0.075	0.397	0.335	0.461	0.283	WHIRLY1
AT1G71260	-0.310	0.247	-0.367	0.185	-0.272	0.413	0.392	0.197	-0.052	0.917	0.136	0.791	WHIRLY2
AT2G02740	-0.015	0.977	0.118	0.825	-0.071	0.909	0.615	0.171	0.151	0.823	0.196	0.804	WHIRLY3
AT3G28910	0.781	0.271	0.807	0.300	0.474	0.629	0.687	0.356	0.863	0.250	0.996	0.207	AtMYB30
AT3G45640	**2.944**	**0.003**	**2.353**	**0.026**	2.114	0.064	1.576	0.133	1.507	0.173	1.397	0.262	AtMPK3
AT4G01370	0.233	0.445	0.208	0.556	0.159	0.701	0.401	0.145	0.299	0.342	0.405	0.196	AtMPK4
AT4G11330	**1.632**	**0.000**	**1.080**	**0.008**	**1.188**	**0.005**	-0.110	0.832	0.013	0.984	0.099	0.890	AtMPK5
AT2G43790	-0.059	0.861	-0.002	0.998	-0.073	0.864	-0.162	0.576	-0.183	0.549	-0.061	0.899	AtMPK6
AT2G30250	**3.292**	**0.000**	**2.621**	**0.000**	**2.766**	**0.000**	**1.423**	**0.000**	**1.588**	**0.000**	**1.777**	**0.000**	WRKY25
AT2G38470	**3.348**	**0.003**	**3.267**	**0.005**	**2.820**	**0.024**	1.926	0.100	1.999	0.099	2.223	0.079	WRKY33

Log_2_ fold changes in transcript abundance (LogFC) were calculated for each transgenic line (*AnAF54*, *PcGCE-7*, *and PcGCE-13*) and tissue (top stem and mid stem) relative to the matching wild-type tissue. LogFC values with FDR < 0.05 are highlighted.

### Mechanism of defense response

It is evident from the RNAseq data that heterologous expression of the microbial hemicellulases resulted in increased transcript abundance of stress and defense-related genes in transgenic arabidopsis plants. Previous studies have noted that the *in planta* expression of certain microbial enzymes, such as β-1,4-xylanase from several *Trichoderma* species, can trigger aspects of plant defense responses such as ethylene biosynthesis, the expression of individual pathogen-related proteins, necrosis-, or hypersensitive response-associated cell death [18–20]. Although our data set does not offer a direct causal explanation for such a phenomenon, it is conceivable that the primary cause can be categorized as (1) the catalytic activity of the microbial hemicellulase, which may generate metabolites or metabolic intermediates that triggered subsequent defense responses, (2) the changes in plant cell wall architecture as the result of the microbial transgene’s catalytic activities which may have compromised the cell wall integrity and relay the subsequent stress responses, or (3) the introduction of a microbial protein, which itself may contain foreign epitopes that are recognized by the plant as pathogenic.

As a consequence of the catalytic activity of a microbial transgene, oligosaccharins can be generated. Oligosaccharins are complex oligosaccharides that are responsible for regulating plant growth as well as elicit defense responses against pathogens [36,37]. Some of the well characterized fungal or plant derived oligosaccharins include chitin, galacturonan, and xyloglucan fragments. Transgenic expression of *Aspergillus niger* polygalacturonase in tobacco and arabidopsis stimulate the release of polygalacturonan which constitutively activate defense responses *in planta* [38]. Interestingly, the PlantGSEA comparison of our core set of genes with data from published studies revealed that the stress response genes enriched in the core gene set strongly and specifically resembled genes differentially regulated in arabidopsis as result of treatment with chitin oligosaccharide [39] (S6 Table). Neither “non-core set” genes nor subsets of genes randomly sampled from all the expressed genes in our study showed this pattern (FDR < 0.05, 1000x resampling, subset size: 655 i.e. the same size as the core gene set). The similarity to chitin oligosaccharide treatment is consistent with our hypothesis of the presence of DAMPs (i.e. oligosaccharins that resulted from the transgene’s catalytic activity) in our transgenic plants. While no evidence has been shown for an elicitation of a defense response, xylan oligosaccharides have been reported to act as an oligosaccharin, with an ability to regulate plant cell growth and expansion [40]. The transgenic plants included in the present study exhibit clear defense-related transcriptome patterns under non-stress conditions, however, additional metabolomic analyses are needed to elucidate the potential of hemicellulases such as PcGCE and AnAF54 for producing DAMPs *in planta*.

Cell wall integrity (CWI) is a prominent form of plant defense, as pathogen invasions typically begin with a perturbation of plant cell walls to enable microbial penetration or enzyme secretion. The loss of CWI can be perceived by the plant via change in mechanistic properties of cell wall or release of DAMP [13]. Arabidopsis employs a number of strategies to sense and respond to altered CWI including sensory proteins [41,42], cell wall degrading enzymes inhibitor [43–45], cell wall reinforcement [46], lignin remodeling [45,47], or pectin modifications [48,49]. Examination of transcript abundance for genes involved in these CWI-related defense mechanisms in our transgenic plants did not yield any expression pattern that was conserved across the transgenes and tissues examined (S7 Table), suggesting that CWI does not play a major role in the defense response patterns that resulted from the presence or catalytic activity of the microbial transgenes investigated in the present study. On a functional level, transgenic expression of other microbial enzymes such as *Aspergillus* acetyl esterase or feruloyl esterase in arabidopsis was shown to alter CWI and to lead to increased sensitivity of plants to actual pathogen infection [16,17].

In addition to the catalytic activities of a microbial transgene, the microbial enzyme itself may act as potential elicitor of the defense response. Enkerli *et al*. (1999) and Sharon *et al*. (1993) have shown that the enzymatic activity of the *Trichoderma* xylanase is not required for the induction of plant stress responses in tobacco by using catalytically inactive mutant variants of a transgene [50,51]. For the hemicellulases included in the present study, the significance of the transgene’s catalytic activity can be validated in the future through transgenic expression of catalytically inactive mutants of these enzymes *in planta*.

### Transcription factors mediate transcriptional defense response patterns in a salicylic acid-dependent manner

We further analyzed the transcription factors in the core set genes with increased transcript abundance, as this may reveal the regulatory mechanism that governs the defense and biotic stress responses in the transgenic plants. Interestingly, of the 22 transcription factors in this category, ten were members of the WRKY transcription superfamily while other transcription factors belonged to the AP2-EREBP, bHLH, bZIP, C2H2, C3H, GRAS, and NAC families (S8 Table). Members of the WRKY transcription factor super family are involved in development and differentiation, but play also key roles in regulating stress responses in arabidopsis [52]. As such, the overrepresentation of WRKY transcription factors in our core set of genes may suggest their relevance to transcriptomic responses of our transgenic plants. Other transcription factor families such as bZIP, MYC, MYB, AP2-EREBP, NAM, ATAF, and NAC can also be relevant for defense responses [53], but were less prevalent in or absent from our core set. For chitin-elicited response in arabidopsis, Libault *et al*. (2007) previously demonstrated a transcription factor representation profile dominated by a variety of different protein families: AP2-EREBP (27), C2H2 (14), MYB (14), and WRKY (14) out of the 118 transcription factors studied [54]. The predominantly WRKY-enriched transcription factor distribution for our transgenic plants suggests that expression of microbial hemicellulases reprograms the transcriptome in a manner that is different from chito-oligosaccharide elicitor-triggered responses.

Members of the WRKY family in the core set were previously shown to be closely linked with salicylic acid. For example, WRKY38 (AT5G22570) was shown to be induced by NPR1 (AT1G64280) in a salicylic acid-dependent manner [55]. Both, WRKY38 and NPR1 are members of the core set. Similarly, the group III WRKY transcription factors, a subgroup of the WRKY transcription factor super family, are induced by salicylic acid in arabidopsis [56]. Members of group III such as WRKY53 (AT4G23810), WRKY54 (AT2G40750), and WRKY70 (AT3G56400) were detected within the core set of genes, and have all been shown to be involved in regulation of plant defense and/or leaf senescence [56,57]. Lastly, SIB1 (AT3G56710) and SIB2 (AT2G41180) are transcriptionally activated by salicylic acid [58], which then interact with WRKY33 (AT2G38470), an important transcription factor in regulating plant defense [59,60]. Intriguingly, both SIB1 and SIB2 are part of the core set. Although WRKY33 itself is not part of the core set, it showed increased transcript abundance in the top stem across all transgenic lines. In addition, several of the kinases that are downstream targets of WRKY33 such as WAK2 (AT1G21270), MPK11 (AT1G01560), and KIN3 (AT2G17220) [61] are all part of the core set, suggesting a close involvement of the WRKY33-related signaling pathway in the defense or biotic stress response in our transgenic lines. Interestingly, NPR1 mediates WRKY70-controlled suppression of the jasmonic acid signaling [57]; further supporting the predominant role of salicylic acid signaling in our transgenic plants. Similarly, WRKY33 can also act as a negative regulator of abscisic acid biosynthesis [61]; its increase in transcript abundance may be another driver to move towards a salicylic acid-dependent response.

Salicylic acid signaling can also be mediated through a NPR1-independent pathway. For example, the salicylic acid-inducible transcription factors WRKY7 (AT4G24240), WRKY15 (AT2G23320), and WRKY25 (AT2G30250) can regulate the salicylic acid-dependent cell death regulator CAD1 (AT5G44070) in a NPR1-independent manner [62]. WRKY7, WRKY15, WRKY25, and CAD1 are part of the core set (S3 Table). Both NPR1-dependent and NPR1-independent responses appeared to be utilized in our transgenic plants, as components as well as WRKY transcription factors from both pathways can be found in the core set. In contrast, only the NPR1-independent signaling was involved in the chitin elicited responses, which may attribute to the differences in transcription factor profiles observed.

Taken together, the enrichment of WRKY transcription factors in the core set provides a strong indication for salicylic acid-dependent responses elicited in the microbial hemicellulase-expressing transgenic arabidopsis investigated in this study.

### The fungal hemicellulase AnAF54 induces genes involved in hemicellulose biosynthesis in young arabidopsis stems

In addition to the core set, genotype specific changes in transcript profiles were also investigated. For each genotype and tissue, genes with differential transcript abundance relative to wild-type were identified. These were then compared between different transgenic arabidopsis lines. Rather than identifying the intersection (core set), genes uniquely reflecting the presence of a particular transgene were selected.

In the *PcGCE* lines, 87 and 165 genes showed a transgene-specific increase in transcript abundance in top and mid stems, respectively; while 895 and 593 genes, were up-regulated in an *AnAF54*-specific manner in top and mid stem samples, respectively (Fig 1A). For genes with transgene-specific reduction in transcript abundance, 100 genes in top stem and 87 genes in mid stem reflected the influence of the investigated glucuronyl esterase (PcGCE), while larger numbers were detected for the specific effect of the studied arabinofuranosidase (AnAF54): 798 genes for top stem and 876 genes for mid stem samples (Fig 1B).

The number of *PcGCE*-specific genes is considerably smaller than the number of *AnAF54*-specific genes. This may be partly due to the inclusion of two *PcGCE* lines compared to one *AnAF54* line. The additional line makes the criteria for *PcGCE*-specific genes more stringent as the changes in transcript abundance need to occur in both lines.

The transgene-specific gene sets were analyzed in similar fashion to that of the core set. Genes with changes in transcript abundance specific to transgenic PcGCE expression are neither enriched in any of the GO slim terminologies nor other categories recognized by PlantGSEA. Intriguingly, 34 *AnAF54*-specific genes with increased transcript abundance in top stem samples were found to be involved in cell wall polysaccharide biosynthesis process (GO: 0070592) (Table 2). Upon closer inspection, major enzymatic activities involved in xylan biosynthesis such as IRX, GUX, GXMT, RWA, as well as their transcriptional regulators such as MYB or SND [63] were all found in this *AnAF54*-specific gene set, suggesting that the expression of the fungal arabinofuranosidase may stimulate xylan biosynthesis during early plant developmental stages. This expression profile substantiates our hypothesis that transgenic expression of microbial enzymes can alter the chemical composition of the cell wall. In this case the microbial arabinofuranosidase is predicted to release arabinose side groups from arabinoxylan, the primary non-cellulosic polysaccharide in secondary cell wall of woody tissues in dicots, thus the plant may produce more xylan to compensate this effect. A similar pattern was not detected in the *PcGCE* lines. However, our previous study [6] shows that PcGCE predominantly affects cell wall structure rather than altering cell wall composition, hence is less likely to have a consistent effect on the cell wall biosynthetic genes.

**Table 2:** Relative fold change of genes involved in call wall biosynthesis (GO: 0070592) in top stem samples of arabidopsis transgenic lines.

Gene Name	*AnAF54*		*PcGCE-7*		*PcGCE-13*			Description
	Log FC	FDR	Log FC	FDR	Log FC	FDR	Protein	
AT1G72990	0.612	0.007	-0.330	0.255	0.235	0.494	BGAL17	hydrolyzes O-glycosyl compounds
AT3G62020	0.814	0.004	-0.263	0.519	0.358	0.378	GLP10	germin-like protein
AT4G17220	1.696	0.000	0.044	0.961	0.783	0.173	MAP70-5	regulates secondary wall patterning in wood cells
AT4G18780	1.110	0.000	-0.296	0.461	0.436	0.264	CESA8	cellulose synthase family
AT1G49950	0.446	0.020	0.100	0.742	0.153	0.608	ATTRB1	Myb domain protein
AT1G08340	1.216	0.005	-0.510	0.385	0.786	0.155		Rho GTPase activating protein
AT1G72220	0.954	0.013	-0.204	0.747	0.535	0.307		RING/U-box superfamily protein
AT4G36890	0.783	0.007	0.047	0.932	0.358	0.386	IRX14	xylose chain elongation
AT5G46340	0.986	0.007	-0.108	0.871	0.577	0.226	RWA1	polysaccharide O-acetylation
AT1G79180	1.287	0.002	-0.194	0.801	0.807	0.142	MYB63	MYB domain protein
AT1G09440	1.238	0.000	-0.168	0.790	0.548	0.273		protein kinase superfamily protein
AT2G03200	1.475	0.002	0.400	0.569	1.085	0.054		aspartyl protease family protein
AT5G60020	1.053	0.002	-0.235	0.667	0.423	0.404	LAC17	laccase activity, lignin biosynthesis
AT4G27435	1.467	0.000	-0.311	0.394	0.593	0.062	DUF1218	
AT5G60720	0.785	0.022	-0.129	0.824	0.392	0.414	DUF547	
AT1G32100	0.775	0.000	-0.172	0.588	0.343	0.222	PRR1	pinoresinol reductase, lignan biosynthesis
AT4G33330	1.156	0.001	-0.114	0.863	0.581	0.218	GUX2	glucuronyltransferase
AT1G62990	0.767	0.001	-0.539	0.046	0.200	0.600	IRX11/KNAT7	homeodomain transcription factor
AT5G15630	1.204	0.001	-0.130	0.842	0.730	0.102	COBL4/IRX6	COBRA family, similar to phytochelatin synthetase
AT4G08160	0.497	0.013	-0.308	0.208	0.266	0.336		putative glycosyl hydrolase family 10 protein (xylanase)
AT1G27440	1.171	0.000	-0.143	0.808	0.584	0.186	GUT2/IRX10	glucuronoxylan biosynthetic process
AT3G56230	1.475	0.001	0.142	0.876	0.254	0.778		BTB/POZ domain-containing protein
AT5G47530	4.023	0.000	0.352	0.870	1.763	0.197		auxin-responsive family protein
AT3G23090	0.760	0.013	-0.401	0.303	0.205	0.688	TPX2	Xklp2 targeting protein
AT5G40020	1.300	0.000	-0.055	0.908	0.545	0.063		Pathogenesis-related thaumatin superfamily protein
AT1G72200	1.424	0.001	-0.330	0.655	0.642	0.308		RING/U-box superfamily protein
AT2G41610	2.670	0.036	-1.100	0.704	1.338	0.538		unknown protein
AT1G33800	1.238	0.009	0.425	0.518	0.842	0.151	GXMT1	glucuronoxylan(GX)-specific 4-O-methyltransferase
AT2G37090	1.201	0.001	-0.105	0.880	0.716	0.122	IRX9	family 43 glycosyl transferase.
AT2G28110	0.920	0.001	-0.567	0.106	0.533	0.151	FRA8/IRX7	glycosyltransferase family 47
AT3G59690	0.943	0.021	0.477	0.360	0.675	0.195	QD13	calmodulin binding
AT4G28500	1.678	0.000	-0.408	0.329	0.676	0.070	NAC073/SND2	NAC domain containing protein 73
AT3G18660	1.328	0.000	-0.517	0.182	0.558	0.168	GUX1	glucuronoxylan biosynthesis
AT5G58980	0.585	0.005	-0.109	0.748	0.095	0.804	NCER3	Neutral/alkaline non-lysosomal ceramidase
AT1G54790	0.956	0.015	0.304	0.593	0.663	0.181		GDSL-motif esterase/acyltransferase/lipase

Log_2_ fold changes in transcript abundance were calculated for each transgenic line (*AnAF54*, *PcGCE-7*, *and PcGCE-13*) and tissue (top stem and mid stem) relative to the matching wild-type tissue.

### The presence of fungal hemicellulases has no major effect on genes that characterize stem development

The growing arabidopsis stem can be described as a gradient of developmental states ranging from an apical region where the young tissue differentiates, followed by regions with rapid cell expansion and maturation, to the basal region where cell wall fortification occurs. Accordingly, we compared the transcriptome profiles obtained from top stem and mid stem for wild-type arabidopsis. This comparison identified 4508 and 4408 genes (FDR < 0.05) with significantly increased and decreased transcript abundance in top stem relative to mid stem in wild-type arabidopsis, respectively, indicating that the top and mid stem samples investigated in the present study reflect clear differences in stem development. A recent study [31] assigned arabidopsis stem segments, based on their actual growth rate, to three developmental categories: “young”, “maximum growth rate”, and “cessation”, and added a fourth category (“old” = stem base) as reference. In the same study, microarray analysis of the different stem categories identified a subset of 4635 differentially expressed genes, and among these eight major clusters of co-expressed genes that characterize the different developmental stages of the arabidopsis stem [31] (S5 Fig). For instance, cluster 1 is defined by genes that are up regulated in “young” stem only; while clusters 3 and 4 comprise genes that are up regulated in regions with cell expansion (“young” and “maximum growth”) and down regulated in “old” stem. Clusters 6, 7, and 8 exhibit expression patterns opposite to clusters 1, 3, and 4, respectively. Most of the genes characterized by Hall and Ellis (2013) [31] as members of the eight clusters were also detected as robustly expressed in our study (84%), and they were found to exhibit distinct differences in their expression between top and mid stem samples (S5A Fig). In our data set, 50–70% of genes in cluster 1, 3, and 4 were identified as genes with significantly increased transcript abundance in the top wild-type stem when compared to the corresponding mid stem samples. Similarly, 40–60% of the genes with cluster 6, 7, and 8 membership showed decreased transcript abundance in the top wild-type stem relative to mid stem in our data set. Such high degree of overlap provided us with confidence that our sample collection strategy corresponded to different stages of plant development. Importantly, joint clustering of our data and data from Hall and Ellis (2013) [31] showed that top stem samples from the present study group with “young” and “maximum growth” stages, while mid stem samples are most similar to older stem segments (“cessation” and “old”) (S5B Fig). Moreover, all top stem and all mid stem samples from transgenic and control plants group together regardless of nature and presence of the transgene (S5B Fig), indicating that the presence of a fungal hemicellulase does not have a major effect on genes that characterize the developmental stage of the stem. Lastly, Ehlting *et al*. (2005) identified ten transcription factors that are up-regulated during stele development [22], seven of these were found in our list of genes with increased transcript abundance in the mid stem. In the same publication, 80 genes were identified as enzymes involved in lignin biosynthesis, which is a hallmark of stem maturation, and 40 were differentially expressed during stem maturation [22]. 31 of these genes were also found in our list of genes with increased transcript abundance in the mid stem. These evidences suggest that the samples collected for top and mid stems correspond well with different plant developmental stages, and that they yield reliable and valuable information on the impact of transgene overexpression on the transcriptome in cell wall engineering.

## Conclusions

Using transcriptome analyses, we were able to systematically demonstrate that heterologous expression of microbial cell wall modifying enzymes in arabidopsis induced defense and stress response pathways. For this, we studied transgenic arabidopsis plants expressing either *Phanerochaete carnosa* glucuronoyl esterase or *Aspergillus nidulans* α-arabinofuranosidase. The observed response is likely to involve the plant-pathogen interaction pathway (KEGG: ATH04626) as well as salicylic acid signaling. At the same time, the expression of fungal transgenes lead to a reduction in transcript abundance for genes involved in antennae proteins of photosystems (KEGG: ATH00196). This result was consistent with the early leaf yellowing phenotypes in the transgenic lines and suggests a tradeoff between investment of plant resources in stress responses and light harvesting. The enrichment of WRKY transcription factors and corresponding upstream/downstream target genes in the transgenic plants were correlated to salicylic acid-mediated stress or defense responses. *A*. *nidulans* arabinofuranosidase expressing plants were further characterized by increased transcript abundance of hemicellulose biosynthesis genes. Subsequent experiments such as metabolomics analyses could help to further elucidate the signaling mechanism and identify potential PAMP candidates produced by the microbial enzymes investigated in this study. An understanding of underlying molecular mechanism as well as regulatory pathways at the omic level is important for critical assessment, improvement, and use of the cell wall engineering strategy described herein. Reducing undesirable phenotypes will lead to improved utilization of biomass and better deployment of lignocellulose applications.

## Supporting information

S1 Fig Leaf yellowing in transgenic arabidopsis lines. (a) Fraction of rosette leaves with yellow color at time point of tissue harvest in wild-type (WT) and transgenic arabidopsis lines (*AnAF54*, *PcGCE-7*, *PcGCE-13*): developmental stage 6.10 [S1]. Images of typical rosettes are shown. (b) Morphology and leaf color of wild-type plants and the AnAF54 line later in development at stage 8.0 [S1]. Scale bar: 5cm. Similar characteristics for the lines *PcGCE-7* and *PcGCE-13* have been published in by Tsai *et al*. (2012) [S2].
(PDF)

S2 Fig Box plot illustrating the magnitude of differences in transcript abundance in transgenic arabidopsis lines for genes constituting the “core set”. The log2 (fold change) between transgenic plants and wild-type plants was calculated for three transgenic lines (*AnAF54*, *PcGCE-7*, and *PcCGE-13*) and two different tissues (top stem, mid stem) each. Data are shown for all genes of the “core set”, i.e. genes that were identified as significantly differentially expressed in every transgenic line and tissue when compared to the respective wild-type control. The core set consists of 467 genes with increased and 188 genes with decreased transcript abundance. For comparison, the average effect across all transgenic lines (all.tg) is also given for mid stem, top stem, as well as both tissues combined.
(PDF)

S3 Fig **Gene ontology (GO) slim term enrichment of the core set genes for molecular function (a) and cellular component (b)**. GO slim terms were annotated using the TAIR bulk GO annotation retrieval tool. The proportion of genes in each GO slim category in the core set of genes was compared to that expected in the background. Asterisks indicate statistical significance (P < 0.05). Black and open bars represent genes with increased and decreased transcript abundance, respectively, while grey bars indicate the gene proportion in each GO slim term for all expressed arabidopsis genes (background).
(PDF)

S4 Fig qRT-PCR validation of transcript abundance patterns for salicylic acid-related genes. Relative transcript abundances of seven salicylic acid-related genes as determined by qRT-PCR was plotted against relative transcript abundances obtained from RNAseq. Open and closed diamonds represent top and mid stem for *AnAF54* (Blue), *PcGCE-7* (Red), and *PcGCE-13* (Green), respectively. Error bars represent standard deviations of the relative expressions.
(PDF)

S5 Fig Expression profiles of developmentally distinct stem tissues. The heat maps show genes with differential transcript abundance based on developmental stage of the stem as determined by kinetic growth profile (n = 4635) [S3]. Data from the present study (red font) were compared to published data (black font), while keeping the topology of eight previously described gene clusters reflecting tissue and developmental stage-specific patterns (grey boxes). Young: top 1 cm of the stem, Max. growth/Max.: stem region with maximum growth rate, Cessation/Ces: stem region with cessation of elongation, Old stem: base of primary stem at rosette [S3], top: top 25% of stem, mid central 50% of stem, WT: wild-type, *AnAF54*, *PcGCE-7*, *PcGCE-13*: transgenic lines overexpressing fungal carbohydrate-active enzymes (present study). (a) Most of the genes identified earlier as characterizing developmentally distinct stem sections were detected in the present study, and among these, 67% were identified as significantly differentially expressed between top and mid stem in non-transformed wild-type plants (ANOVA contrast, FDR < 0.05). (b) Clustering reveals a distinct grouping of all top stem samples regardless of transgene. The same is observed for mid stem samples. Top stem samples from this study cluster with profiles from young stem and stem regions with the largest growth rate, while mid stem samples group with stem segment characterized by cessation of growth. Thin blue lines represent boundaries of the gene clusters 1–8 described in Hall and Ellis (2013) [S3]. Data from Hall and Ellis (2013) represent estimates (log scale) relative to all treatment classes [S3], data from this study are centered log transformed cpm values (counts per million), averages per tissue are given.
(TIF)

S1 Table qRT-PCR primers. (XLSX)

S2 Table Relative fold change of all Genes, all genotypes, and all tissues. (XLSX)

S3 Table Relative fold change of the core set genes. (XLSX)

S4 Table Relative fold change of genes involved in plant-pathogen interaction (KEGG ath04626) (a) and in the photosynthesis—antenna proteins (KEGG ath00196) (b).
(XLSX)

S5 Table Relative fold change of genes involved in hormonal biosynthesis and signaling. (XLSX)

S6 Table Relative fold change of genes in the core set that overlapped with genes responding to chitin elicitor. (XLSX)

S7 Table Relative fold change of genes involved in cell wall integrity (CWI). (XLSX)

S8 Table Relative fold change of transcription factors in the core set genes. (XLSX)

S1 File References for supporting information. (PDF)
