# Supplementary figures and images for: Transgenic expression of fungal accessory hemicellulases in Arabidopsis thaliana triggers transcriptional patterns related to biotic stress and defense response
Source: PLoS One. 2017 Mar 2;12(3):e0173094. doi: 10.1371/journal.pone.0173094 (PMC5333852; doi:10.1371/journal.pone.0173094)

(a)

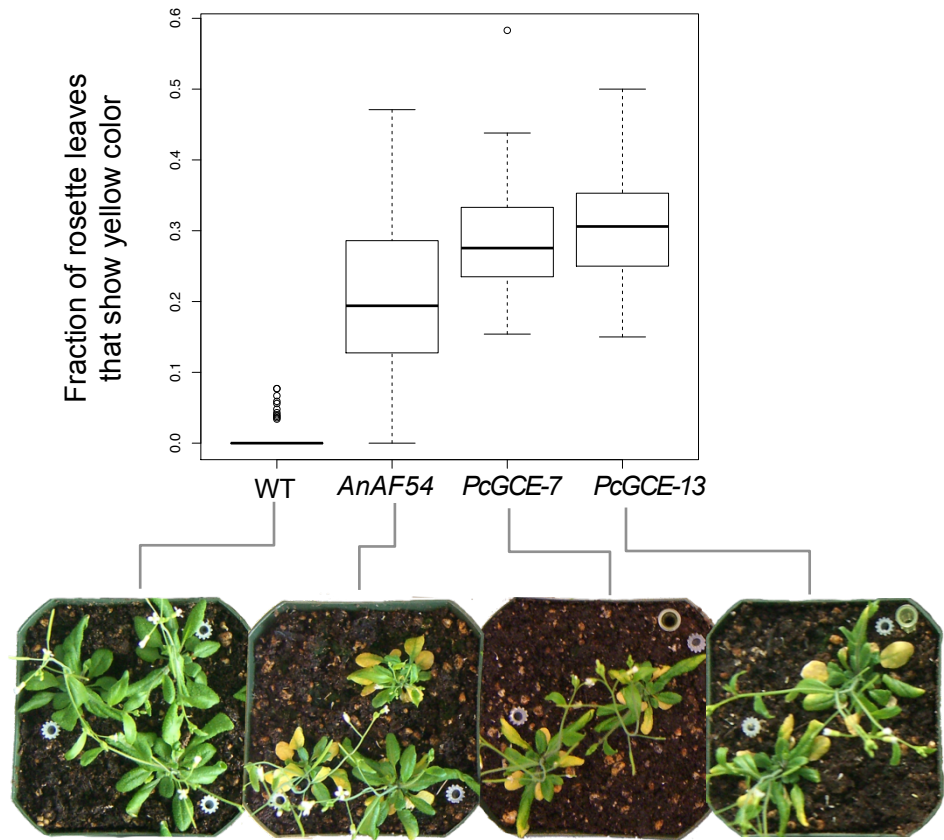

(b)

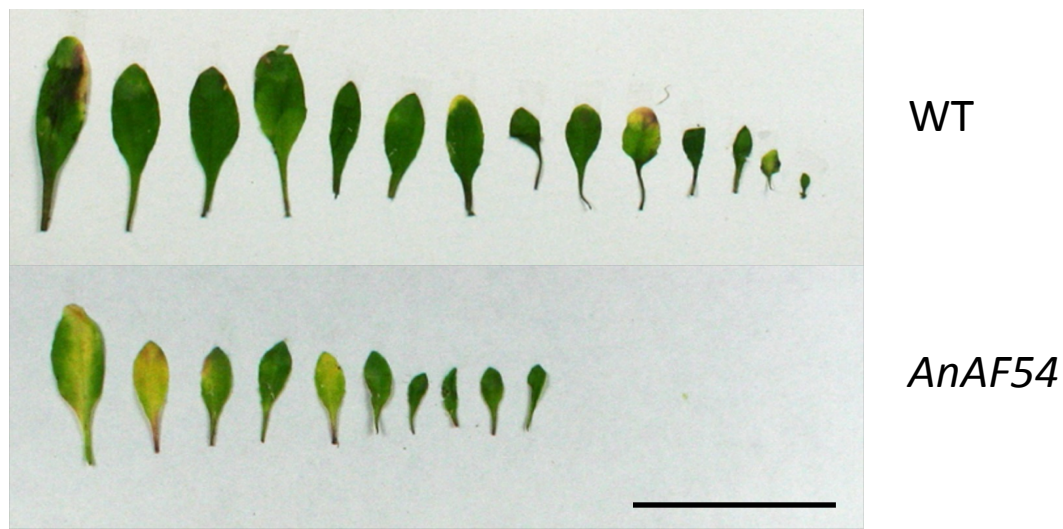

Supplement: S1 Fig — (a) Fraction of rosette leaves with yellow color at time point of tissue harvest in wild-type (WT) and transgenic arabidopsis lines (AnAF54, PcGCE-7, PcGCE-13): developmental stage 6.10 [S1]. Images of typical rosettes are shown. (b) Morphology and leaf color of wild-type plants and the AnAF54 line later in development at stage 8.0 [S1]. Scale bar: 5cm. Similar characteristics for the lines PcGCE-7 and PcGCE-13 have been published in by Tsai et al. (2012) [S2]. (PDF) [file pone.0173094.s001.pdf]

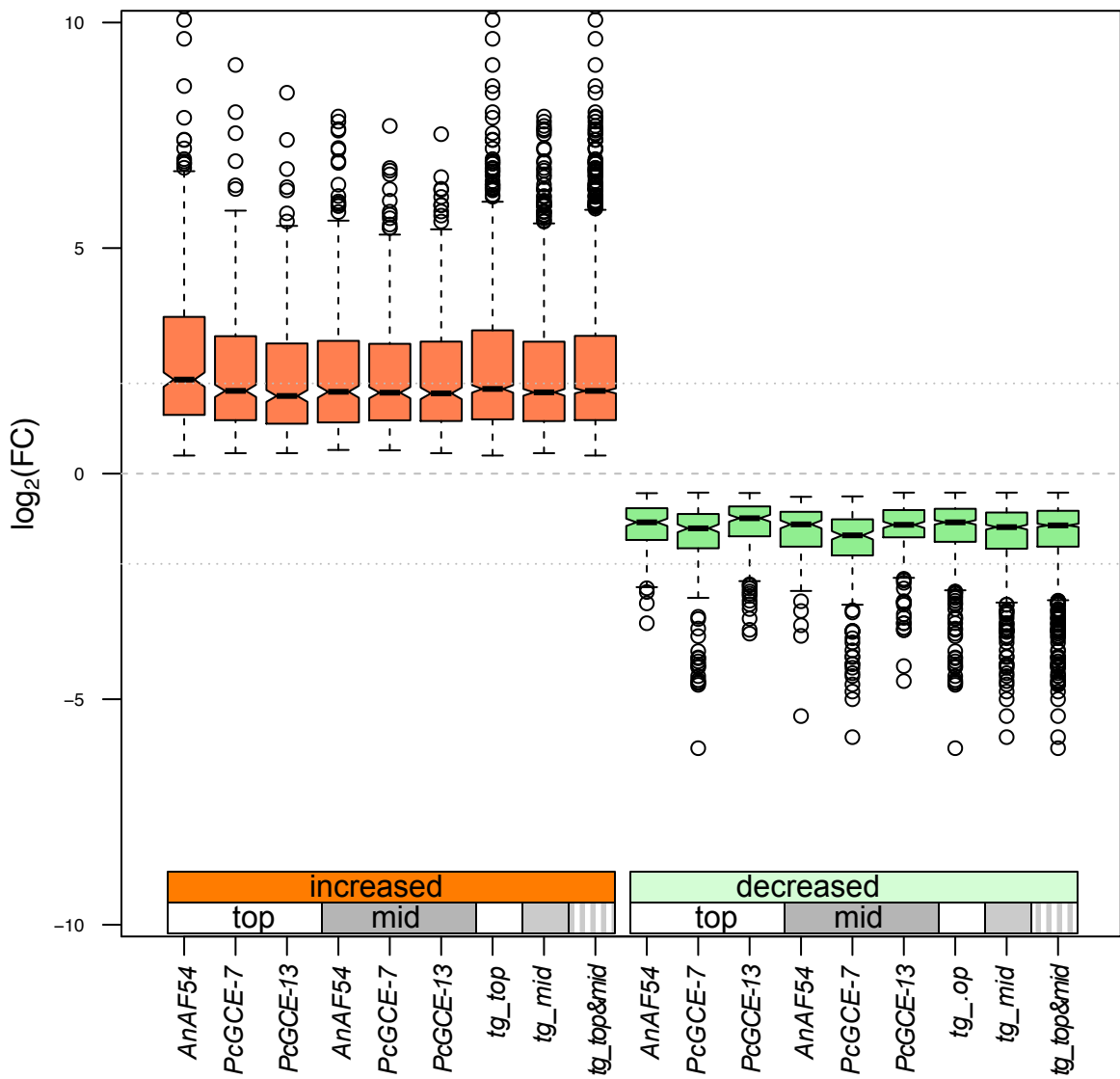

Supplement: S2 Fig — The log2 (fold change) between transgenic plants and wild-type plants was calculated for three transgenic lines (AnAF54, PcGCE-7, and PcCGE-13) and two different tissues (top stem, mid stem) each. Data are shown for all genes of the “core set”, i.e. genes that were identified as significantly differentially expressed in every transgenic line and tissue when compared to the respective wild-type control. The core set consists of 467 genes with increased and 188 genes with decreased transcript abundance. For comparison, the average effect across all transgenic lines (all.tg) is also given for mid stem, top stem, as well as both tissues combined. (PDF) [file pone.0173094.s002.pdf]

(a)

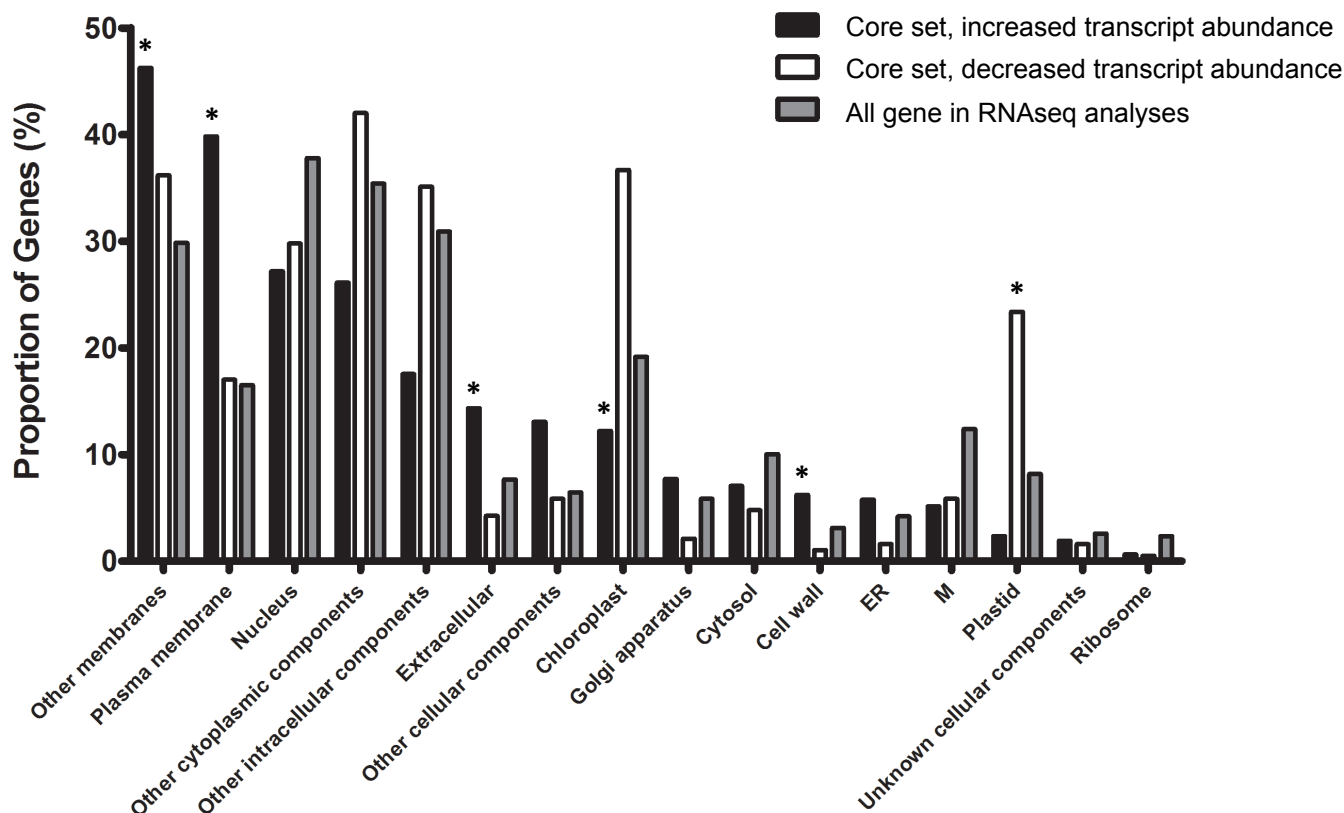

(b)

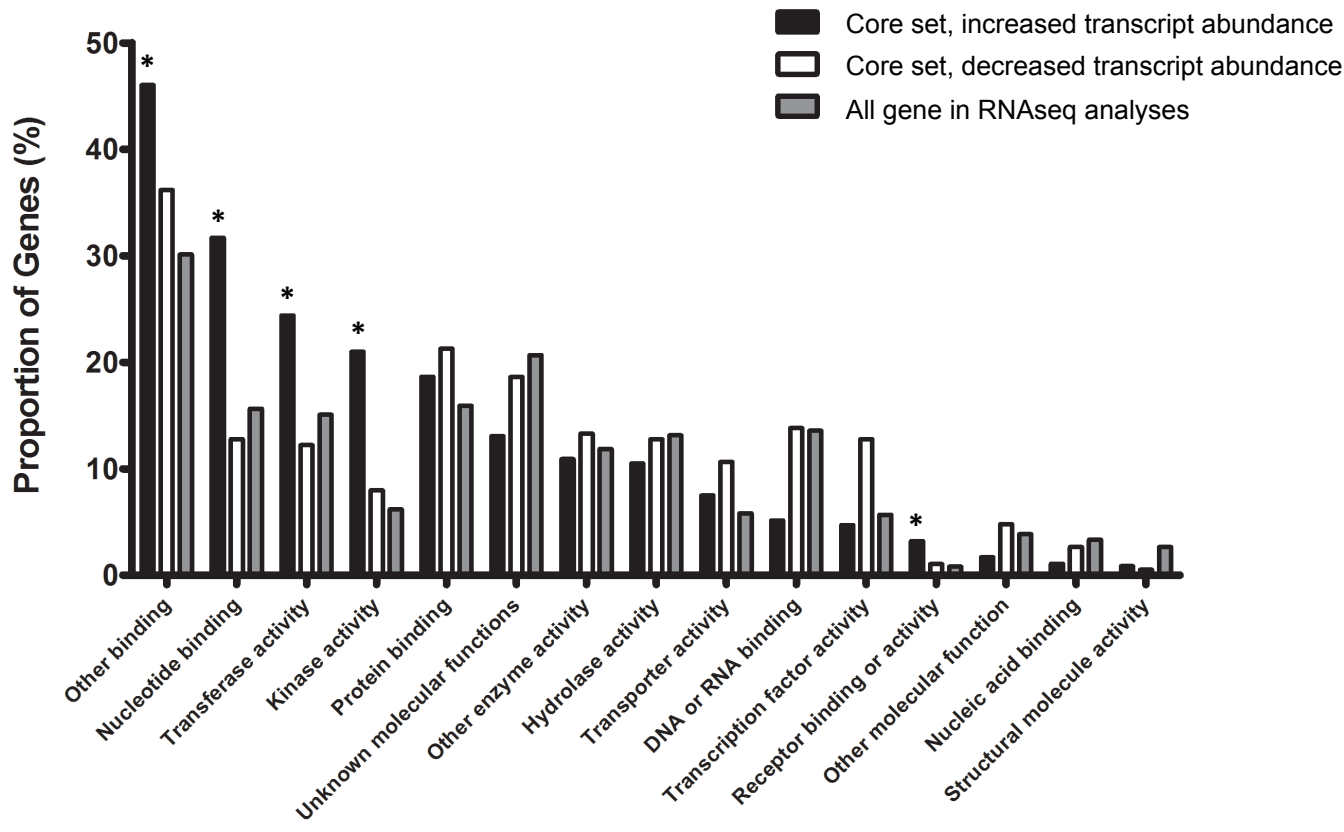

Supplement: S3 Fig — Gene ontology (GO) slim term enrichment of the core set genes for molecular function (a) and cellular component (b). GO slim terms were annotated using the TAIR bulk GO annotation retrieval tool. The proportion of genes in each GO slim category in the core set of genes was compared to that expected in the background. Asterisks indicate statistical significance (P < 0.05). Black and open bars represent genes with increased and decreased transcript abundance, respectively, while grey bars indicate the gene proportion in each GO slim term for all expressed arabidopsis genes (background). (PDF) [file pone.0173094.s003.pdf]

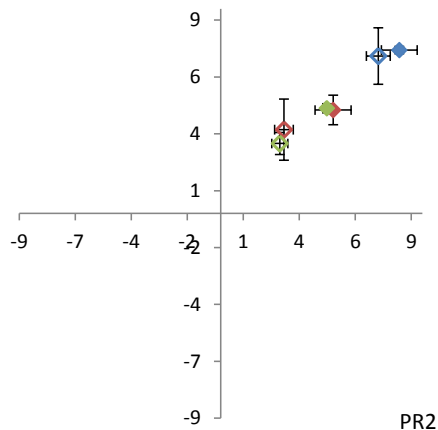

PR2

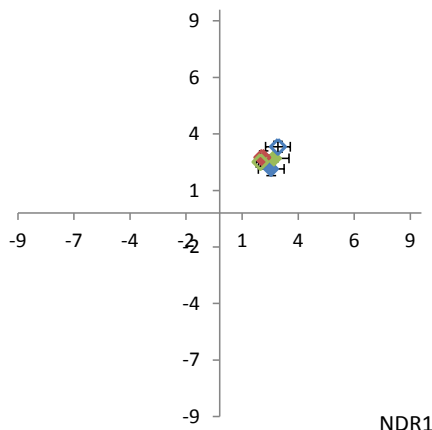

NDR1

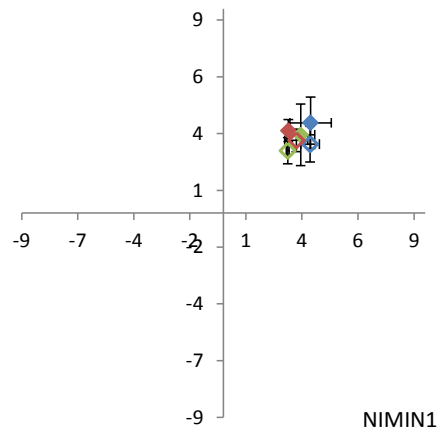

NIMIN1

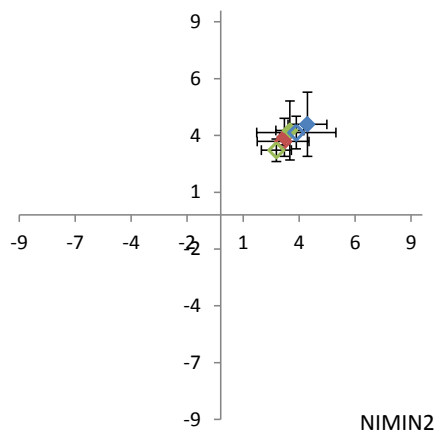

NIMIN2

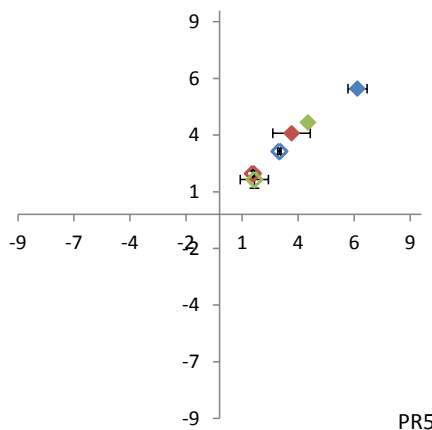

PR5

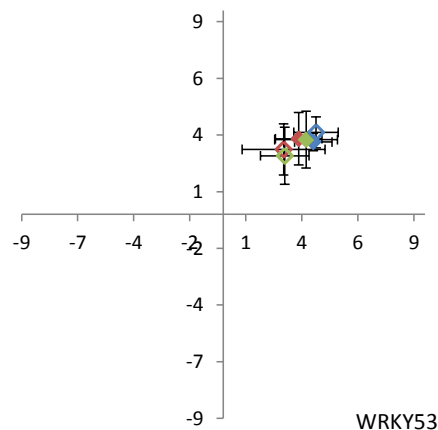

WRKY53

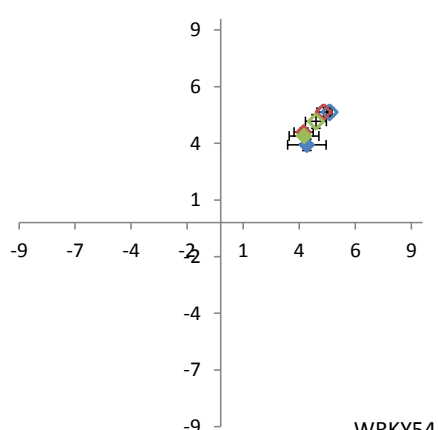

WRKY54

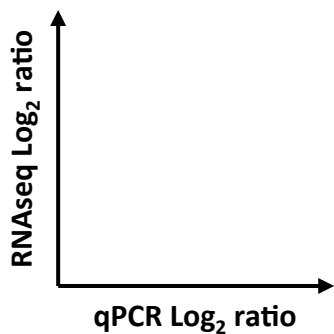

- ◆ *AnAF54\_Mid*
- ◆ *PcGCE-7\_Mid*
- ◆ *PcGCE-13\_Mid*
- ◇ *AnAF54\_Top*
- ◇ *PcGCE-7\_Top*
- ◇ *PcGCE-13\_Top*

Supplement: S4 Fig — Relative transcript abundances of seven salicylic acid-related genes as determined by qRT-PCR was plotted against relative transcript abundances obtained from RNAseq. Open and closed diamonds represent top and mid stem for AnAF54 (Blue), PcGCE-7 (Red), and PcGCE-13 (Green), respectively. Error bars represent standard deviations of the relative expressions. (PDF) [file pone.0173094.s004.pdf]

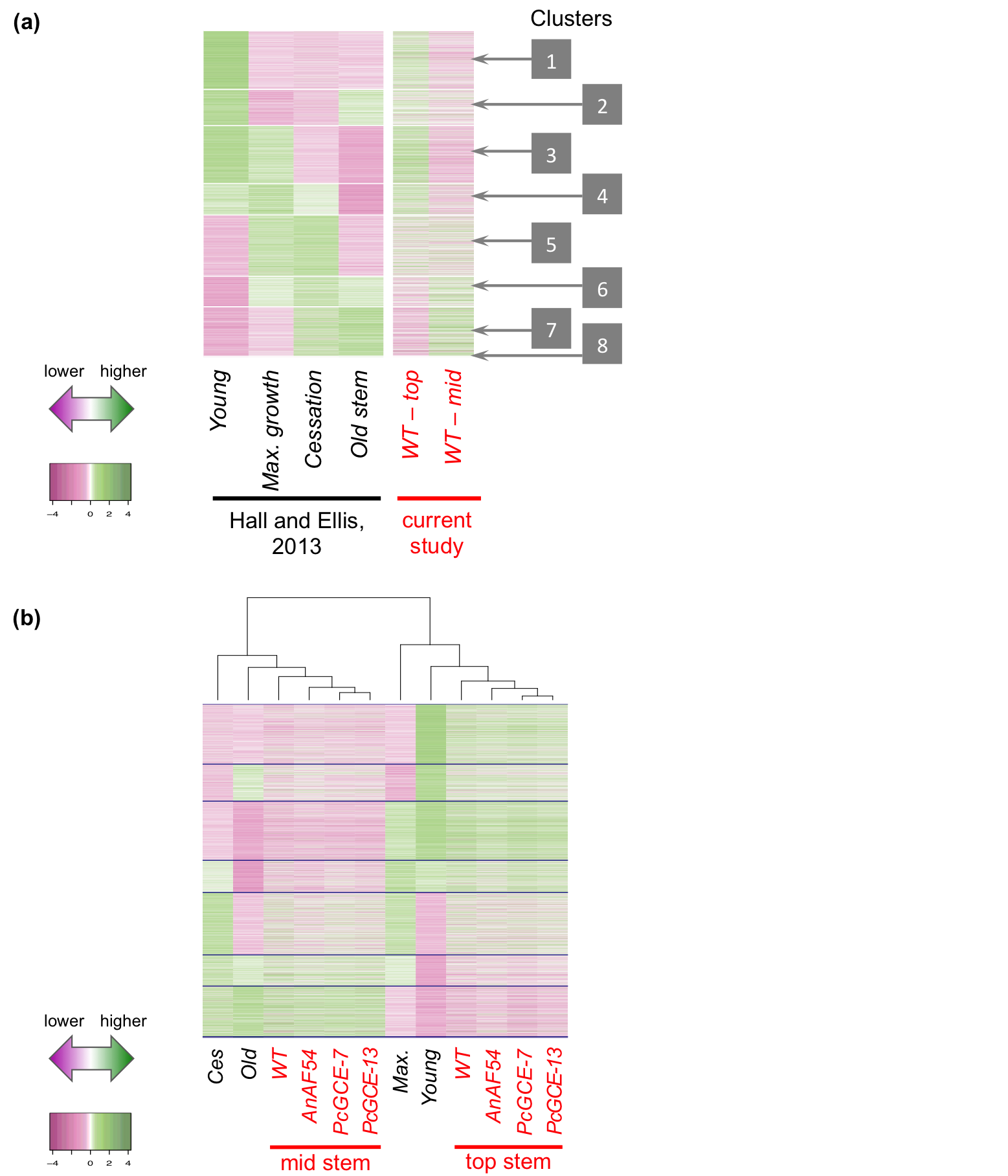

Supplement: S5 Fig — The heat maps show genes with differential transcript abundance based on developmental stage of the stem as determined by kinetic growth profile (n = 4635) [S3]. Data from the present study (red font) were compared to published data (black font), while keeping the topology of eight previously described gene clusters reflecting tissue and developmental stage-specific patterns (grey boxes). Young: top 1 cm of the stem, Max. growth/Max.: stem region with maximum growth rate, Cessation/Ces: stem region with cessation of elongation, Old stem: base of primary stem at rosette [S3], top: top 25% of stem, mid central 50% of stem, WT: wild-type, AnAF54, PcGCE-7, PcGCE-13: transgenic lines overexpressing fungal carbohydrate-active enzymes (present study). (a) Most of the genes identified earlier as characterizing developmentally distinct stem sections were detected in the present study, and among these, 67% were identified as significantly differentially expressed between top and mid stem in non-transformed wild-type plants (ANOVA contrast, FDR < 0.05). (b) Clustering reveals a distinct grouping of all top stem samples regardless of transgene. The same is observed for mid stem samples. Top stem samples from this study cluster with profiles from young stem and stem regions with the largest growth rate, while mid stem samples group with stem segment characterized by cessation of growth. Thin blue lines represent boundaries of the gene clusters 1–8 described in Hall and Ellis (2013) [S3]. Data from Hall and Ellis (2013) represent estimates (log scale) relative to all treatment classes [S3], data from this study are centered log transformed cpm values (counts per million), averages per tissue are given. (TIF) [file pone.0173094.s005.tif]
