# Supplementary material for: Transgenic expression of fungal accessory hemicellulases in Arabidopsis thaliana triggers transcriptional patterns related to biotic stress and defense response
Source: PLoS One. 2017 Mar 2;12(3):e0173094. doi: 10.1371/journal.pone.0173094 (PMC5333852; doi:10.1371/journal.pone.0173094)
Supplement: S1 File — (PDF) [file pone.0173094.s014.pdf]

References – supporting information

S1. Boyes DC, Zayed AM, Ascenzi R, McCaskill AJ, Hoffman NE, Davis KR, et al. Growth stage-based phenotypic analysis of Arabidopsis: a model for high throughput functional genomics in plants. Plant Cell. 2001;13: 1499–510.

S2. Tsai AY-L, Canam T, Gorzsás A, Mellerowicz EJ, Campbell MM, Master ER. Constitutive expression of a fungal glucuronoyl esterase in Arabidopsis reveals altered cell wall composition and structure. Plant Biotechnol J. 2012;10: 1077–87.

S3. Hall H, Ellis B. Transcriptional programming during cell wall maturation in the expanding Arabidopsis stem. BMC Plant Biol. 2013;13: 14.

S4. Vlot AC, Dempsey DA, Klessig DF. Salicylic Acid, a multifaceted hormone to combat disease. Annu Rev Phytopathol. 2009;47: 177–206. doi:10.1146/annurev.phyto.050908.135202

S5. Merilo E, Jalakas P, Laanemets K, Mohammadi O, Hõrak H, Kollist H, et al. Abscisic acid transport and homeostasis in the context of stomatal regulation. Mol Plant. 2015;8: 1321–1333. doi:10.1016/j.molp.2015.06.006

S6. Qin F, Shinozaki K, Yamaguchi-Shinozaki K. Achievements and challenges in understanding plant abiotic stress responses and tolerance. Plant Cell Physiol. 2011;52: 1569–1582. doi:10.1093/pcp/pcr106

S7. Caspi R, Altman T, Billington R, Dreher K, Foerster H, Fulcher CA, et al. The MetaCyc database of metabolic pathways and enzymes and the BioCyc collection of Pathway/Genome Databases. Nucleic Acids Res. 2014;42: 471–480. doi:10.1093/nar/gkt1103

S8. Sasaki Y, Asamizu E, Shibata D, Nakamura Y, Kaneko T, Awai K, et al. Monitoring of methyl jasmonate-responsive genes in Arabidopsis by cDNA macroarray: self-activation of jasmonic acid biosynthesis and crosstalk with other phytohormone signaling pathways. DNA Res. 2001;8: 153–161. doi:10.1093/dnares/8.4.153

S9. Li Z, Peng J, Wen X, Guo H. Gene network analysis and functional studies of senescence-associated genes reveal novel regulators of Arabidopsis leaf senescence. J Integr Plant Biol. 2012;54: 526–39.
